# A Case of Poisoning from the External Application of the Cocculus Indicus

**Published:** 1852-04

**Authors:** William B. Thompson

**Affiliations:** Sen. House Surgeon, Emigrant’s Hospital, Ward’s Island


*A Case of Poisoning from the external application of the Cocculus Indicus.* Reported by William B. Thompson, Sen. House Surgeon, Emigrant's Hospital, Ward's Island.

Bridget Maddon—aged 6—native of Ireland—blue eyes and fair complexion, was admitted into the surgical division, accompanied by a sister and brother, on the evening of Feb. 3d, 1852. Disease—porrigo of the scalp, infected with vermin.

The nurse was directed, after cutting the hair close, to wash their heads with an infusion of Delphinia, (the house prescription.) Not having the article at the time, the apothecary sent an infusion of Cocculus Indicus, (used in the hospital for the same purpose,) made by displacement—a pound to three gallons of dilute alcohol—six ounces of which was used for the three children.

Half an hour after its application, I was sent for to see Bridget, whom I found laboring under tetanic spasms with the pupils contracted to their smallest diameter. As the spasm abated, the pupils became dilated to their fullest extent, and again contracting as the spasm returned. By touching the eyelids, the spasm could be produced at pleasure; this was repeated until all present became fully satisfied the difficulty was seated in the excito-motory system, and was the result of irritation rather than inflammation. The case was treated as one of idiopathic tetanus—with counter-irritation, warm baths and antispasmodics; but the attacks continued to increase in frequency and force, until the patient sank about midnight of the same day. A post-mortem examination was made of the body thirty hours after death. The viscera, brain, and its appendages, were minutely examined, and all found to be in a healthy condition. The nature of the case is confirmed by the younger sister, aged four, being attacked in the same manner and exhibiting the same symptoms as she (Bridget) did. Being in the Ward at the time, a warm bath was ordered, into which she was placed as soon as the first spasm was over; a mustard plaster was applied over the abdomen and to the legs and feet as high as the knee; injections of the tincture of assafetida were thrown into the rectum and a few drops administered by the mouth every hour. By persevering with these means the spasms subsided gradually after an elapse of three hours from the commencement of the attack. On the morning of the 4th inst. the patient's body and arms were again covered with an eruption resembling scarlatina, which gradually faded away during the day.

